# Selection of Lactic Acid Bacteria (LAB) Antagonizing *Vibrio parahaemolyticus*: The Pathogen of Acute Hepatopancreatic Necrosis Disease (AHPND) in Whiteleg Shrimp (*Penaeus vannamei*)

**DOI:** 10.3390/biology8040091

**Published:** 2019-12-01

**Authors:** Linh Nguyen Thi Truc, Ai Trinh Ngoc, To Tran Thi Hong, Tuu Nguyen Thanh, Huong Huynh Kim, Long Pham Kim, Giang Huynh Truong, Phu Truong Quoc, Tinh Nguyen Thi Ngoc

**Affiliations:** 1 Tra Vinh University, 126, Nguyen Thien Thanh, Tra Vinh city, Tra Vinh Province 87000, Vietnam; truclinh@tvu.edu.vn (L.N.T.T.); ngocai@tvu.edu.vn (A.T.N.); tthongto@tvu.edu.vn (T.T.T.H.); hkhuong77@tvu.edu.vn (H.H.K.); kimlongpham@gmail.com (L.P.K.); 2 Department of Aquatic Pathology, College of Aquaculture and Fisheries, Can Tho University, 3/2 street, Ninh Kieu, Can Tho City 94000, Vietnam; htgiang@ctu.edu.vn (G.H.T.); tqphu@ctu.edu.vn (P.T.Q.); 3 Research Institute for Aquaculture No. 2, District 1, Ho Chi Minh City 71000, Vietnam; ntngoctinh@yahoo.co.uk

**Keywords:** AHPND, LAB, *Vibrio parahaemolyticus*, whiteleg shrimp

## Abstract

Acute hepatopancreatic necrosis disease (AHPND) has recently emerged as a serious disease of cultured shrimp. A total of 19 lactic acid bacteria (LAB) strains isolated from shrimp samples were characterized based on morphological characteristics, biochemical tests, sequencing analysis, and their ability to antagonize *Vibrio parahaemolyticus*, which causes AHPND in whiteleg shrimp. Results from the agar well diffusion method indicated that 3 out of 19 isolated LAB strains showed the highest antagonizing ability against AHPND *V. parahaemolyticus* strain with an inhibition zone diameter ranging from 18 to 20 mm. Experiments where shrimps were given feed supplemented with these LAB strains and challenged with AHPND strain showed high survival rates (approximately 80.0%), which were not significantly different as compared to those recorded in the negative control treatment (86.6%), but significantly different to those recorded in the positive control treatment (40.6%) after 16 days of the experiment. However, the histological images of shrimp hepatopancreas indicated that the infection rate significantly reduced from 60.0% to 11.1% in shrimps fed with LAB-supplemented feeds and challenged with AHPND *V. parahaemolyticus* strain as compared to those in the positive control treatment. A polymerase chain reaction (PCR) and 16S rRNA gene sequencing confirmed the identification of LAB strain. These results can be applied in further experiments to investigate the ability of *L. plantarum* in preventing AHPND in intensively cultured whiteleg shrimp.

## 1. Introduction

In Vietnam, brackish-water shrimps are considered as one of the most important food products in the international market, estimating at almost 20% of the overall aquaculture commodities in trade. Presently, rapidly developing industrialization has led to the deterioration of the environment, especially the aquatic environment. The development of diseases in aquaculture industry has increased since the involvement of the intensification of aquaculture activities [1]. One of the crises is the proliferation of pathogenic bacteria known as *Vibrio* species, which have been frequently associated with mortalities both in hatcheries and grow out ponds [2,3].

According to a prior study [4], the causative agent of acute hepatopancreatic necrosis disease (AHPND) is a unique strain of *Vibrio parahaemolyticus* that produces toxins responsible for the loss of more than $1 billion per year of marine shrimp farming. This disease has led to high mortality of cultured marine shrimps at Soc Trang, Bac Lieu, Tra Vinh, and Ca Mau provinces in Vietnam. In 2012, the total affected area in the Mekong Delta was 39,000 ha [5]. Recent research has indicated that AHPND has led to serious loss in some countries [6]. Some methods have been suggested to prevent AHPND in shrimps such as using chemicals, antibiotics, or biological products.

However, using chemicals and antibiotics might cause environmental contamination and antibiotic resistance of the pathogenic bacteria. Moreover, chemical or antibiotic residuals are one of the criteria that plays a vital role in exporting agricultural products. Therefore, biological methods that are considered as the best methods for preventing diseases are used not only for controlling the density of pathogenic bacteria but also for ensuring food safety and, more importantly, as a friendly environmental method [7].

Probiotics, known as an alternative to antibiotics and antimicrobial drugs, have been suggested to control and manage disease problems in the aquaculture industry. Probiotics are considered as live micro-organisms and if used in adequate amounts can confer a health benefit to the host [8]. Lactic acid bacteria (LAB) are probiotics for human and animals, and play a vital role in stimulating digestion and preventing harmful bacteria [9]. Presently, LAB are being selected to supplement in aquaculture feed because of its benefits such as removing pathogens [10,11], providing nutrition and enzyme for digestion [12], and enhancing the immune system of animals [10,13,14,15]. The combination between couple of strains of *L. Casei* and *S.cerevisiae* or triple of strains from *L. casei*, *S. cerevisiae*, and *R. palustris* revealed the better inhibiting AHPND for shrimp culture [16]. The maintenance population diversified useful bacteria and caused a decreased risk of AHPND in shrimp ponds [17].

During the fermentation process, LAB can produce organic acid that can limit the growth of pathogenic bacteria through the effect of organic acid on the surface of the bacteria [18,19,20]. According to a prior study [21], *Lactobacillus plantarum* have a resistance to AHPND-causing *V. parahaemolyticus* as proven by agar well diffusion method. *V. harveyi* infection was prevented by using *L. plantarum* MRO3.12 [22]. From previous researches, a hypothesis that LAB could have the ability to antagonize AHPND-causing *V. parahaemolyticus* was stipulated.

The present study focused on the phenotypic and biochemical characterization of the 19 LAB strains and 16S rRNA gene sequence analysis carried out to provide a reference point to evaluate their resistance towards AHPND-causing *V. parahaemolyticus.*

## 2. Materials and Methods

### 2.1. Isolation and Identification of LAB Isolates

#### 2.1.1. Isolation of LAB Isolates

A total of 50 shrimp samples were collected from extensive and improved extensive shrimp farms in Ben Tre and Tra Vinh provinces, Vietnam. Shrimp surface was sterilized with 70% ethanol. Thereafter, the intestinal tract of shrimps was removed and placed in test tubes containing 3 mL of sterilized 0.85% NaCl solution. Shrimp intestine was crushed by means of a glass rod to form a homogenate solution which was left for 5 min to settle down. After the settlement, 1 mL of the supernatant was collected and mixed with de Man Rogosa Sharpe (MRS, Merck, Darmstadt, Germany) broth supplemented with 1.5% NaCl. These tubes were incubated at 28 °C for 48 h. The suspension was then diluted with sterilized 0.85% NaCl solution at 10^−1^, 10^−2^, and 10^−3^ dilutions, respectively. 50 µL of each dilution was spread on MRS agar supplemented with 1.5% NaCl and 1% CaCO_3_ and incubated at 28 °C for 48 h. Colonies with opaque, creamy, and smooth round characteristics showing CaCO_3_ resolution on MRS agar were picked up to test for physiological (gram staining, spore forming) and biological (production of oxidase and catalase) characteristics. Colonies that were gram-positive, non-spore forming, oxidase-negative, and catalase-negative were identified as LAB.

#### 2.1.2. Determination of Antagonizing Ability Against AHPND *V. parahaemolyticus* Strain by the Well Diffusion Method

The antagonizing ability of LAB isolates against AHPND *V. parahaemolyticus* strain using the agar well diffusion method was determined following the guidelines described by [23]. AHPND *V. parahaemolyticus* strain used in this experiment was previously isolated from AHPND infected shrimps and stored at the Laboratory of Pathology Department, Can Tho University. *V. parahaemolyticus* was briefly recovered and cultured in Tryptic Soy Broth (TSB, Merck, Darmstadt, Germany) supplemented with 1.5% NaCl at 28 °C for 24 h. Subsequently, sterilized swabs were dipped into culture and spread on nutrient agar (NA, Merck, Darmstadt, Germany) plates supplemented with 1.5% NaCl. Thereafter, 4 wells were punched on each agar plate with each LAB isolate cultured in 5 mL MRS broth supplemented with 1.5% NaCl at 28 °C for 48 h. Subsequently, 1 mL of the culture was collected and put into 1.5 mL Eppendorf tube and centrifuged at 10,000 rpm for 20 min. Next, 50 µL of the supernatant was then collected and placed into the wells made on agar plates with three replications for each isolate. The fourth well was filled with autoclaved distilled water and used as the control well. The agar plates were subsequently incubated at 28 °C for 24 h. After incubation, the diameters of inhibition zone were measured and an antagonizing ability of LAB towards AHPND *V. parahaemolyticus* strain determined based on the protocol described by [23].

#### 2.1.3. Microbiological Identification Using the API-50 CHL System

Fermentation of carbohydrates was determined using API 50 CHL, a standardized system, consisting of 50 biochemical tests for the study of carbohydrate metabolism by micro-organisms. API 50 CHL was used in conjunction with API 50 CHL medium for the identification of *Lactobacillus* and related genera strips. Three LAB isolated showing high antagonizing ability towards AHPND *V. parahaemolyticus* strain were identified using the API-50 CHL kit according to the manufacturer’s instructions (Biomerieux, Marcy l’Etoile, France) [24].

Further, 5 mL of the LAB cultures and 10 mL of distilled water were introduced into the API 50 CHL medium. The setup system was then incubated at 30 °C for 24 and 48 h, after the wells were filled with the LAB suspensions by the line marked with the addition of paraffin oil. The color changes were recorded as results of API strips reaction. Numerical profiles of strains were identified adding positive values in the indicative table. The API LAB PLUS database (Bio Merieux, Marcy l’Etoile, France) was used for the interpretation of the results.

#### 2.1.4. Molecular Identification

Further identification was examined by amplifying and sequencing the partial 16S rRNA gene for the LAB isolates. Genomic DNA was extracted as described by [25]. A single colony was transferred to a 5 mL microtube with 1 mL of liquid medium from which the isolate was originally picked up. The cultures were incubated for three to five days at 28 °C with shaking at 180 rpm. Bacterial cells from these cultures were collected by centrifugation. Bacterial 16S rDNA fragments were amplified by polymerase chain reaction (PCR) using the 27F primer (5′-AGAGTTTGATCCTGGCTCAG-3′) [26] and 1492R primer (5′-TACGGTTACCTTGTTACGACTT-3′) [27]. PCR conditions were as follows: initial denaturation at 95 °C for 5 min, 30 cycles of 95 °C for 30 s, 53 °C for 30 s, and 72 °C for 1.5 min, and a final extension of 5 min at 72 °C. The PCR products were thereafter sent to FIRST-Base base-Asia, (Singapore). The NCBI BLAST (National Center for Biotechnology Information) database was used to analyze sequence homology for extracted 16S rRNA gene sequences. In general, the sequences of genes were mapped with nucleic acid sequences from GenBank database which showed high similar results. The 16S rRNA gene sequences were compared with those from the type strains available in NCBI (accession number: MK6113341.1, MK369235.1, MH681600.1) using the basic local alignment search tool (BLAST) [28].

### 2.2. Determination of the Effect of LAB-Supplemented Feed on AHPND Resistance of Whiteleg Shrimps

#### 2.2.1. Experimental Setup

Whiteleg shrimps at a size of about 1 g showing white spot disease-negative [29] and AHPND-negative [30] were used in this experiment. AHPND *V. Parahaemolyticus* strain stored at the Laboratory of Fisheries Faculty of Can Tho University was recovered and cultured in nutrient broth (NB, Merck, Darmstadt, Germany) supplemented with 1.5% NaCl and incubated at 28 °C for 24 h. Thereafter, bacterial density was determined by measuring the optical density at 610 nm.

Commercial feed (CP Brand name) with 40% protein was used to feed the experimental shrimps. Each LAB isolate was cultured in MRS broth supplemented with 1.5% NaCl at 28°C for 48h, the LAB density was identified by optical density (OD) at 1 ± 0.025 with 610 nm wavelength after 48 h, approximately 10^9^ CFU/mL LAB. The cultured solution was later centrifuged at 7000 rpm for 5 min and then sterilized saline solution aim to clean and to dilute the biomass the bacterial strains to 10^9^ CFU/mL. An aliquot of 10 mL solution of each bacterial strain was added into 100 g shrimp feed (10^8^ CFU/g) covered by squid oil. The shrimp feed was packed and labelled and stored at 4 °C before feeding. The feed was preserved and feed the shrimps within seven days.

Checking the LAB density: Take 1 g feed merging into 9 mL sterilized saline solution and strong shake until the feed was totally separated, then, diluting the solution with 10^2^, 10^3^, and 10^4^ CFU/mL, respectively. Taking 0.05 mL of each to spread onto the MRS agar petries, which was already added 1.5% NaCl, and then samples were cultured at 28 °C in 48 h. The grown colony quantity used to calculate the LAB density by applying the formula:

$$Density\;of\;LAB\;\left(CFU/g\right)=\;\frac{number\;of\;colony\;\times \;diluted\;solution\;}{0.05\;mL\times feed\;sample\;weight}$$

The pellet was washed thrice with sterilized 0.85% NaCl solution and mixed with shrimp feed afterwards. The density of LAB was adjusted to 10^8^ CFU per gram of feed. In the treatments where shrimps were given LAB supplemented feed, shrimps were fed with *L. plantarum* (VPL1), *Lactobacillus fermentum* (VPL2)*, and Pediococcus pentosaceus* (VPL3), respectively, for 20 days before challenging with AHPND *V. parahaemolyticus* strain. Challenge experiments were carried out following the protocol described by [31]. Shrimps were immersed in *V. parahaemolyticus* suspension at a density of 2 × 10^7^ CFU/mL for 15 min. After that, the challenging shrimps were placed in tanks with sea water containing *V. parahaemolyticus* at a density of 10^6^ CFU/mL. In the negative control treatment, shrimps were immersed in sterilized TSB medium supplemented with 1.5% NaCl and later placed in tanks without bacteria supplementation.

#### 2.2.2. Experimental Design

A total of 30 shrimps were placed in a glass tank containing 20 L of 20 ppt seawater. Experiments were done with eight treatments and three replications/treatment (total 24 glass tanks). In treatment one, shrimps were given normal feed without LAB supplementation (negative control, NC). In treatment two, shrimps were given normal feed without LAB supplementation and challenged with AHPND *V. Parahaemolyticus* (positive control, PC). In treatments three, four, and five, shrimps were given feed supplemented with *L. plantarum* (L1), *L. fermentum* (L2), and *P. pentosaceus* (L3), respectively. In treatments six, seven, and eight, shrimps were given feed supplemented with *L. plantarum* (VPL1), *L. fermentum* (VPL2), and *P. pentosaceus* (VPL3), respectively, and challenged with AHPND *V. parahaemolyticus* (Table 1). Challenge method was done following the protocol described by [31]. Shrimps were challenged with AHPND *V. parahaemolyticus* at 10^6^ CFU/mL and fed four times daily and water was exchanged at 30% after three days of challenge and further exchanged at 30% daily. The temperature was kept at 28 °C, pH at 7.5–8.0, nitrite below 4 mg/L, ammonia below 0.13 mg/L and alkalinity at 110–120 mg CaCO_3_/L. Water quality parameters were observed daily using Sera test kits (Sera, Germany) to maintain suitable conditions for shrimp culture.

#### 2.2.3. Parameters

Density of LAB and *Vibrio* in shrimp gut was determined based on a counting method described in a previous work [32], just before the challenge and every three days after challenge. Briefly, three shrimp individuals were collected randomly from each tank. Samples of shrimp gut were collected in sterile conditions, weighed, and milled in 0.85% NaCl solution. Thereafter, the suspension was diluted to different concentrations and 20 µL of each dilution was dropped on MRS agar plates supplemented with 1.5% NaCl and on Thiosulfate Citrate Bile Salts Sucrose (TCBS, Merck, Darmstadt, Germany) agar plates to enumerate LAB and *Vibrio*, respectively. The MRS agar and TCBS agar plates were further incubated at 28 °C for 48 and 24 h, respectively. The densities of the bacteria were determined by counting the colonies growing on agar plates using this formula:

Density of bacteria (CFU/g) = (number of colonies × dilution/V)/M

where *V* is volume of the suspension placed on MRS/TCBS agar plate (mL) and *M* is the weight of the shrimp gut (g).

Survival rate of shrimps after 16 days of challenge was calculated using the formula given as:

$$Survival\;rate\;\left(\%\right)\;=\;\frac{surviving\;shrimps\;after\;challenge\;}{total\;shrimps\;in\;tank}\times 100\%\;$$

Shrimps were observed for clinical signs after challenge. Moreover, three shrimps per tank were randomly collected to examine for histopathology change of hepatopancreas. Histological analysis was performed following the protocol described in a past work [33]. Shrimp hepatopancreas were fixed in Davidson’s solution for 48 h and placed in 70% ethanol solution. Subsequently, samples were passed through ethyl alcohol 70%, 80%, 95%, 100%, and xylene solution. Samples were then cut into pieces with 5 µm thickness. Subsequently, the slide samples were stained with Hematoxylin and Eosin (H and E) and the infected and healthy shrimp tissues identified under microscope at 10X and 40X magnification and labelled for further data analysis.

### 2.3. Statistical Analysis

SPSS software of 22.0 version was used to compare the mean between different treatments and one-way analysis of variance (ANOVA) used to analyze the data. Duncan multiple-comparison’s test was used to determine the significant difference among treatments at a 0.05 significance level.

## 3. Results

### 3.1. Isolation of LAB

In this study, a total of 19 LAB isolates, including 8 strains from Tra Vinh province and 11 strains from Ben Tre province, were isolated from 50 shrimp samples. For these isolates, colony’s diameters ranged from 1 to 2 mm and colony’s appearance were opaque, creamy, smooth, and round. For physiological characteristics, these isolates were cocci and rod-shape, gram-positive, and non-spore forming. For biochemical characteristics, all 19 isolates were CaCO_3_-dissolving, oxidase-negative, and catalase-negative (Table 2).

### 3.2. Antagonizing Ability of LAB Isolates Against AHPND-V. parahaemolyticus

**Figure 1:**
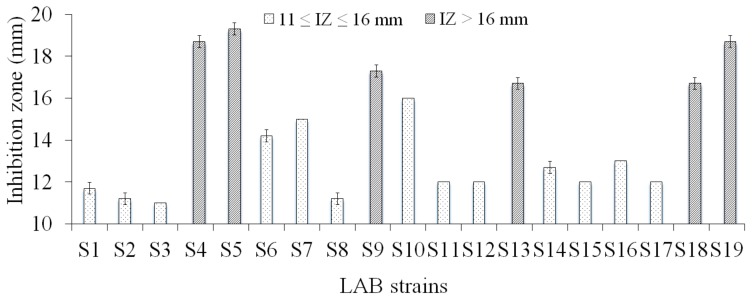
Diameter of inhibition zones by LAB isolates against *V. parahaemolyticus* (IZ: Inhibition Zone). Arrow bar indicated SD = standard deviation with three replications.

Among the 19 tested isolates, three isolates (S4, S5, and S19) were found to inhibit the growth of AHPND *V. parahaemolyticus* strain with the diameters of inhibition zone ranging from 18.0 to 20.0 mm, while the other 16 isolates showed intermediate inhibition with the diameters of clearance zone ranging from 11.0 to 15.0 mm, respectively (Figure 1).

### 3.3. Identification of LAB Isolates Showing High Antagonizing Ability Against AHPND V. Parahaemolyticus

#### 3.3.1. Identification of LAB Isolates by API50 CHL System

For the biochemical identification of three LAB isolates with diameters of an inhibition zone larger than 18.0 mm (S4, S5 and S19 isolates), the API 50 CHL system was used. These isolates were identified as *L. plantarum, L. fermentum*, and *P. pentosaceus*, respectively.

#### 3.3.2. Identification of LAB Isolates by 16S rRNA Gene Sequencing

From the results of APICHL system test, one of the three LAB strains that can prevent AHPND in whiteleg shrimp (*P. vannamei*) was selected for 16S rRNA sequencing. The results indicated that the length of the PCR product was 993bp (GenBank accession number: KX369235.1). The results retrieved were 16S rRNA gene sequences of LAB indicating high similarity (99.78%) which confirmed the presence of *L. plantarum*.

### 3.4. Effect of in-feed LAB Addition on the Resistance to AHPND in White Leg Shrimps

#### 3.4.1. Dynamics of LAB Density in the Gut of Experimental Shrimps

Table 3 indicated that treatments without LAB addition in feed showed no presence of LAB in the shrimp gut. In contrast, treatments with LAB in-feed addition and without AHPND *V. parahaemolyticus* challenge showed a regular increase in LAB density. After 16 days, LAB densities in all the treatments were significantly higher than day 0 (*P* < 0.05). 

With respect to treatments with LAB in-feed addition and shrimps challenged with AHPND strain, LAB density also remained and increased in the shrimp gut. However, LAB density showed a big fluctuation at different sampling times. In treatments VPL2 and VPL3, LAB density tended to decrease and reached the lowest value at 4.2 × 10^5^ and 2.5 × 10^5^ CFU/g, respectively, after eight days of challenge. Although LAB density in these treatments increased afterwards, it was significantly lower than day zero. However, LAB density in VPL1 treatment after 12 days of challenge (6.9 × 10^5^ CFU/g) was not significantly different as compared to day 0 (6.6 × 10^5^ CFU/g).

#### 3.4.2. Vibrio Density in the Gut of Experimental Shrimps

In the treatment without LAB addition and AHPND strain challenge (negative control), *Vibrio* density in the shrimp gut did not significantly change during the experiment (ranging from 17.3 to 40.7 × 10^5^ CFU/g). In the treatment without LAB addition and with AHPND strain challenge (positive control), *Vibrio* density in the shrimp gut was significantly increased after the challenge and finally, in the treatments with LAB addition and without challenge, *Vibrio* density in shrimp gut significantly decreased after four days of LAB feeding, with exception of treatment L3, which showed no significant change in *Vibrio* density. Among treatments with LAB addition and AHPND strain challenge, treatments VPL1 and VPL2 showed a significant decrease of *Vibrio* density in shrimp gut after 12 days of LAB feeding. However, treatment VPL3 did not show any significant change in *Vibrio* density (Table 4). Generally, LAB supplementation in feed can reduce *Vibrio* density in the gut of whiteleg shrimps.

#### 3.4.3. Effect of LAB In-Feed Addition on the Survival of White-Leg Shrimps Challenged with AHPND Strain

**Figure 2:**
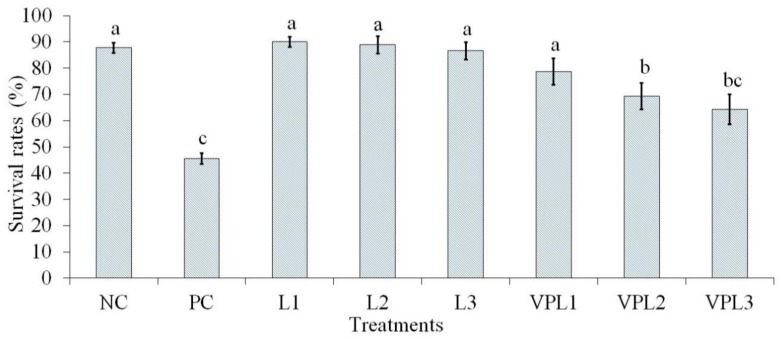
Survival rates of whiteleg shrimps fed dietary supplemented LAB. PC: Positive control; NC: Negative control; L1: *Lactobacillus plantarum*, L2: *Lactobacillus fermentum*, and L3: *Pediococcus pentosaceus*; VP: AHPND strain challenge; treatments with different letters were significantly different (*P* < 0.05). Arrow bar indicated SD = standard deviation with three replications.

After several hours of post challenge with *V. parahaemolyticus* at 10^6^ CFU/mL, the experimental shrimps stopped feeding and a few shrimps died at 6 h post infection. Figure 2 shows the average survival rate of shrimps fed on diet with LAB supplementation. After 16 days of treatment, there were significant differences (*P* < 0.05) in survival rate between the treated group and non-treated group (PC group). Shrimps which were given feed supplemented with a LAB strain and without challenge showed the greatest survival rate (86.60–90.90%), compared to shrimps in the positive control (40.57%). Obviously, shrimp survival in one of the treatments with a LAB strain supplementation and challenge with AHPND strain (VPL1) appeared to be enhanced compared to those in the other two groups (VPL2 and VPL3).

### 3.5. Histopathology of Shrimp Hepatopancreas

**Figure 3:**
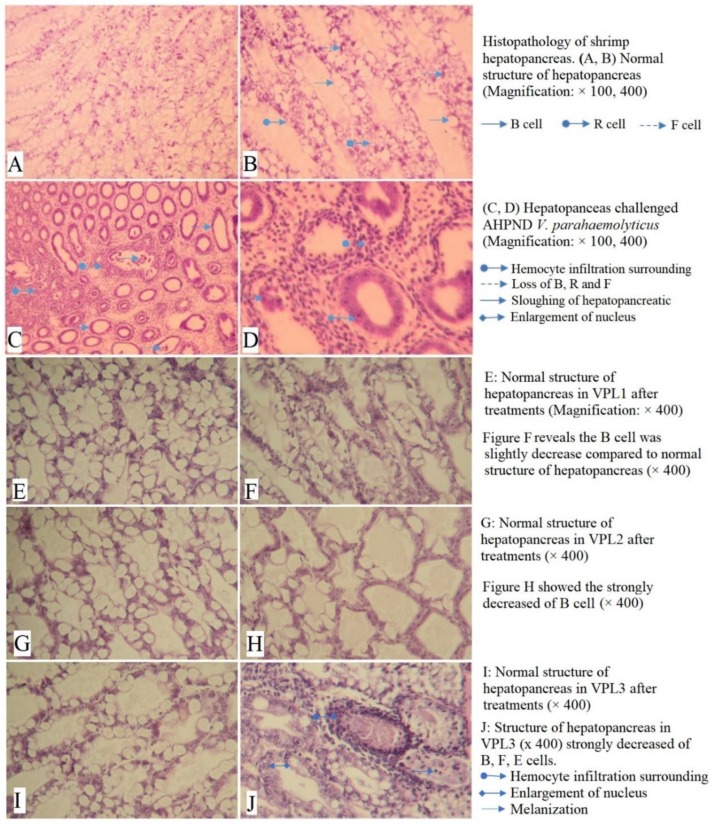
Histopathology of shrimp hepatopancreas.

The histopathology of shrimp hepatopancreas indicated that shrimps without challenge showed a normal structure of hepatopancreas with a high number of B cells and R cells (Figure 3A,B). In contrast, H and E sections from infected shrimps showed the histopathological effects of bacterial infection in hepatopancreatic tissues. Shrimps infected with the AHPND strain indicated a change in hepatopancreas structure (Figure 3C,D). However, the level of changes were different among treatments, particularly treatments PC, VPL1, VPL2, and VPL3, which indicated 66.7, 11.1, 33.3, and 44.4% of samples that showed signs of AHPND infection in hepatopancreas, respectively, after a four-day challenge. On the other hand, in the treatments where shrimps were given feed supplemented with *L. plantarum* and challenged with AHPND strain, the proportion of diseased shrimps was significantly reduced, and no observation of histopathology was found in shrimps in these treatments. There were no significant differences between these treatments and negative control treatment at the end of the experiment.

## 4. Discussion

It is important to provide healthy shrimps with higher production and probiotics has a great deal of potential [34]. In aquaculture, several methods have been applied to enhance the health condition of farmed animals, including reproducing specific disease-resistant shrimps [35], specific pathogen-free shrimps [36], and the use of probiotics [13,37,38]. Probiotics can improve the survival, immunity, and disease resistance of aquatic animals [13,37]. Probiotics can demonstrate several modes of action, including producing inhibitory compounds, competing for chemicals, or an available energy or adhesion site, as well as enhancing the host immune response.

In the present study, a total of 19 LAB strains were isolated from diseased shrimps and identified by morphological, biochemical, and molecular techniques. Subsequently, experiments to investigate the effect of these LAB strains on AHPND resistance in whiteleg shrimps were conducted. The results of these experiments indicated that the use of three LAB strains (*L. plantarum*, *L. fermentum*, and *P. pentosaceus*) supplemented in feed can reduce the mortality rate in shrimps challenged with AHPND strain. However, the level of protection was strain-dependent. Generally, LAB bacteria can limit the growth of *Vibrio* species using different mechanisms and have the ability to produce acetaldehyde, H_2_O_2_, diacetyl, CO_2_, multisugar, and bacteriocins, which can limit bacterial growth [19,39,40]. Additionally, LAB can exhibit maintaining a voltage of cell membrane, including transporting, reducing internal pH, and a host of metabolism functions. [41]. *V. parahaemolyticus* is inhibited at pH less than 5.5; no *V. parahaemolyticus* strains can grow at low pH levels at which LAB strains can grow [42].

Bacteria, which reside in the gut of aquatic animals, play an important role in the digestion functions of cultured animals, and the gut microbe can affect cultured animals in several aspects, including nutrition and immune response [43] through the links among epithelium cells, the reduction of excretion stimulation, and the effect due to bacterial inflammation.

LAB strains provided a good growth and clinging in the gut of experimental shrimps and can maintain a very high density in shrimps fed LAB supplementation, with and without AHPND *V. parahaemolyticus* challenge. The previous study demonstrated that the weight of shrimps significantly gained from 4.32 ± 0.03 to 4.48 ± 0.20 g/shrimp after supplemented *L. plantarum* MRO3.12 [22]. The density of LAB in the shrimp gut was lowest at day three to six in shrimps given feed with LAB supplementation and challenged with *V. parahaemolyticus* due to competition of an AHPND *V. parahaemolyticus* in the gut of challenged shrimps. This resulted in a decrease of LAB in the gut. However, the density of LAB increased, indicating a positive growth in the shrimp gut, particularly the growth of *L. plantarum*. This result is in line with a past report [44] that indicated that *Lactobacillus* can bring several benefits to cultured animals since they can cling on cell walls, thus preventing the growth of pathogenic bacteria and balancing the microbial composition in the gut. This study showed that *L. plantarum* strain exhibited the ability to grow and establish itself in the gut of experimental shrimps. 

Several studies have reported on the benefits of *Lactobacillus* in resisting some pathogens in fish. *L. plantarum*, *Lactobacillus*, and *Carnobacterium* strains isolated from rotifer can limit the density of some *Vibrio* species which causes disease in flounder larvae in the shrimp gut [45,46]. Moreover, *L. plantarum* is one of the important species used in fermenting several foods and can also release antibacterial substances that prevent some pathogens, such as plantaricin [47]. This study indicated that the LAB strains isolated from shrimp samples, particularly *L. plantarum*, have the ability of limiting the growth of *Vibrio* in shrimp gut.

In this study, LAB strains can provide higher survival rate of challenged shrimps fed on diet supplemented with LAB compared to that of challenged shrimps fed on diet without LAB supplementation. Similarly, another study [48] reported that survival of aquaculture animals increased as they fed on diets supplemented with *Lactobacillus* sp. Yet another study [23] indicated that the survival of black tiger shrimps improved from 20% to 87% as shrimps were fed diet supplemented with *Lactobacillus* and challenged with *V. alginolyticus* for 10 days. It was reported that the diet mixed with *L. plantarum* enhanced the immune system, as well as the digestion ability of whiteleg shrimps [45], resulting in higher survival rates of challenged shrimps. 

AHPND is a serious disease that has caused damages and significant financial losses to the global shrimp aquaculture industry and can cause up to 100% mortality. According to histopathological images of this study, infected shrimps showed principal signs of AHPND [49], including changes of hepatopancreatic structures, lack of B, F, and R cells, sloughing of hepatopancreatic cells, presence of melanization, and hemocytic infiltration surrounding hepatopancreatic tubules (Figure 3C,D). Shrimps were less affected by AHPND pathogen at challenge treatments with *L. plantarum* supplementation (11.11% mortality) compared to challenge treatments without LAB supplementation (66.67% mortality). The result indicated that *L. plantarum* could be used as a measure to prevent AHPND in shrimps since it can improve the ability of shrimps to resist AHPND infection. 

The study mainly conducted on a practice for antagonizing *V. parahaemolyticus*. The pathogen of AHPND in whiteleg shrimp (*Penaeus vannamei*) was studied in tank condition but with some remarkable improvements: (1) LAB strains were isolated by authors from the experiments; (2) the identified LAB strains were isolated from wild shrimp sources which were actually collected from the local rearing ponds in Tra Vinh and Ben Tre provinces (Vietnam) without using probiotics, drugs, and chemicals during the culture period; and (3) the results already demonstrated that the ability to antagonize AHPND up to 20 mm diameter of inhibition zones is higher than previous studies. 

## 5. Conclusions

*L. plantarum* strain isolated in this study can provide several benefits for shrimps in AHPND *V. parahaemolyticus* challenge conditions, including the reduction of *Vibrio* density in shrimp gut, conferring higher survival rates of shrimps, and a stronger overall resistance to AHPND. The application of *L. plantarum* strain in intensive shrimp ponds should be studied in future research. It would be better if the LAB strain is applied for experiments on an actual field scale and combined with studies of a shrimp immunologist.

## Figures and Tables

**Table 1:** Description of experimental design.

S/No	Treatments	Feed with LAB (10^8^ CFU/mL)	AHPND *V. Parahaemolyticus* (10^6^ CFU/mL)
1	NC	No	No
2	PC	No	Yes
3	L1	*Lactobacillus fermentum*	No
4	L2	*Pediococcus pentosaceus*	No
5	L3	*Pediococcus pentosaceus*	No
6	VPL1	*Lactobacillus fermentum*	Yes
7	VPL2	*Pediococcus pentosaceus*	Yes
8	VPL3	*Pediococcus pentosaceus*	Yes

Note: Positive control; NC: Negative control; L1: *Lactobacillus plantarum*, L2: *Lactobacillus fermentum*, and L3: *Pediococcus pentosaceus*; and VP: AHPND strain challenge.

**Table 2:** Morphological, physiological, and biochemical characteristics of LAB isolated in Tra Vinh and Ben Tre, Vietnam.

Province	Shrimp Samples (10 g/individual)	Total Isolates	Morphological Characteristics		Physiological Characteristics			Biochemical Characteristics		
			Colony Diameter (mm)	Colony Shape	Cell Shape	Gram-Staining	Spore-Forming	Catalase	Oxidase	Lactic Acid Production (CaCO_3_ Dissolving)
Tra Vinh	Shrimp	8	1–1.5	Opaque, creamy, smooth round	Cocci, rod	+	−	−	−	8
Ben Tre	Shrimp	11	1–2	Opaque, creamy, smooth round	Cocci, rod	+	−	−	−	11

Note: + is positive; − is negative.

**Table 3:** Dynamics of LAB density in the gut of experimental shrimps (10^5^ CFU/g).

NT	Day 0	Day 4	Day 8	Day 12	Day 16
NC	0.00^ạ^	0.00 ^a^	0.00 ^a^	0.00 ^a^	0.00 ^a^
PC	0.00 ^a^	0.00 ^a^	0.00 ^a^	0.00 ^a^	0.00 ^a^
L1	0.00 ^c^	2.40 ± 0.53 ^c^	8.0 ± 1.60 ^b^	9.30 ± 0.38 ^a^	9.00 ± 0.07 ^a^
L2	0.00 ^c^	9.00 ± 0.50 ^b^	10.00 ± 1.60 ^b^	16.00 ± 13.00 ^a^	15.00 ± 1.10 ^a^
L3	0.00 ^c^	3.90 ± 0.80 ^b^	3.30 ± 0.83 ^b^	4.80 ± 0.25 ^b^	8.80 ± 1.10 ^a^
VPL1	6.60 ± 0.47 ^a^	5.40 ± 1.50 ^b^	5.30 ± 0.15 ^b^	6.90 ± 1.30 ^a^	7.70 ± 0.15 ^a^
VPL2	8.40 ± 0.08 ^a^	4.60 ± 0.85 ^c^	4.20 ± 0.53 ^c^	5.50 ± 0.96 ^b^	5.10 ± 0.90 ^b^
VPL3	5.20 ± 1.40 ^b^	9.80 ± 7.50 ^a^	2.50 ± 1.00 ^d^	3.80 ± 2.20 ^c^	3.80 ± 2.10 ^c^

Notes: PC: Positive control; NC: Negative control; L1: *Lactobacillus plantarum*, L2: *Lactobacillus fermentum*, and L3: *Pediococcus pentosaceus;* VP: AHPND strain challenge; treatments with different superscripts were significantly different (*P* < 0.05). Different letters (a, b, c, and d) in the numbers on each row indicate significant differences (Duncan’s Test and Tukey Test).

**Table 4:** Dynamics of *Vibrio* density in the gut of experimental shrimps (10^5^ CFU/g).

NT	Day 0	Day 4	Day 8	Day 12	Day 16
NC	31.00 ±26.30 ^a^	40.70 ± 23.70 ^a^	24.50 ± 2.40 ^a^	17.30 ± 12.90 ^a^	33.80 ± 29.90 ^a^
PC	16.90 ± 10.50 ^b^	53.30 ± 39.70 ^a^	52.70 ± 41.50 ^a^	65.30 ± 14.80 ^a^	56.30 ± 34.60 ^a^
L1	18.90 ±7.30 ^a^	6.10 ± 3.20 ^b^	3.40 ± 2.50 ^b^	2.20 ± 1.99 ^b^	0.08 ± 0.01 ^b^
L2	25.50 ± 17.00 ^a^	10.70 ± 8.30 ^ab^	4.20 ± 3.40 ^b^	0.40 ± 0.20 ^b^	0.26 ± 0.20 ^b^
L3	14.00 ± 10.00 ^a^	4.20 ± 4.00 ^a^	2.90 ± 0.60 ^a^	2.80 ± 1.60 ^a^	2.30 ± 1.60 ^a^
VPL1	47.00 ± 12.30 ^a^	36.90 ± 29.70 ^a^	11.50 ± 8.20 ^b^	10.60 ± 6.40 ^b^	9.50 ± 5.60 ^b^
VPL2	40.70 ± 37.70 ^a^	17.50 ± 8.20 ^ab^	15.80 ± 7.90 ^ab^	13.50 ± 4.80 ^ab^	10.57 ± 0.74 ^b^
VPL3	40.70 ± 32.50 ^a^	30.00 ± 26.70 ^a^	23.80 ± 21.00 ^a^	17.10 ± 11.90 ^a^	14.40 ± 3.80 ^a^

Notes: PC: Positive control; NC: Negative control; L1: *Lactobacillus plantarum*, L2: *Lactobacillus fermentum*, and L3: *Pediococcus pentosaceus*; VP: AHPND strain challenge; treatments with different superscripts were significantly different (*P* < 0.05). Different letters (a and b) in the numbers on each row indicate significant differences (Duncan’s Test and Tukey Test).
